# Identifying and Predicting Novelty in Microbiome Studies

**DOI:** 10.1128/mBio.02099-18

**Published:** 2018-11-13

**Authors:** Xiaoquan Su, Gongchao Jing, Daniel McDonald, Honglei Wang, Zengbin Wang, Antonio Gonzalez, Zheng Sun, Shi Huang, Jose Navas, Rob Knight, Jian Xu

**Affiliations:** a Single-Cell Center, CAS Key Laboratory of Biofuels and Shandong Key Laboratory of Energy Genetics, Qingdao Institute of BioEnergy and Bioprocess Technology, Chinese Academy of Sciences, Qingdao, Shandong, China; b Department of Pediatrics, University of California San Diego, La Jolla, California, USA; c Department of Computer Science & Engineering, University of California San Diego, La Jolla, California, USA; d Department of Bioengineering, University of California San Diego, La Jolla, California, USA; e Center for Microbiome Innovation, University of California San Diego, La Jolla, California, USA; f Laboratory for Marine Biology and Biotechnology, Qingdao National Laboratory for Marine Science and Technology, Qingdao, Shandong, China; g University of Chinese Academy of Sciences, Beijing, China; University of Hawaii at Manoa; University of Hawaii at Manoa; DOE Joint Genome Institute

**Keywords:** microbiome, search, novelty, data mining, bioinformatics, community similarity, database search, microbial ecology, microbiome, microbiome novelty

## Abstract

With the expansion of microbiome sequencing globally, a key challenge is to relate new microbiome samples to the existing space of microbiome samples. Here, we present Microbiome Search Engine (MSE), which enables the rapid search of query microbiome samples against a large, well-curated reference microbiome database organized by taxonomic similarity at the whole-microbiome level. Tracking the microbiome novelty score (MNS) over 8 years of microbiome depositions based on searching in more than 100,000 global 16S rRNA gene amplicon samples, we detected that the structural novelty of human microbiomes is approaching saturation and likely bounded, whereas that in environmental habitats remains 5 times higher. Via the microbiome focus index (MFI), which is derived from the MNS and microbiome attention score (MAS), we objectively track and compare the structural-novelty and attracted-attention scores of individual microbiome samples and projects, and we predict future trends in the field. For example, marine and indoor environments and mother-baby interactions are likely to receive disproportionate additional attention based on recent trends. Therefore, MNS, MAS, and MFI are proposed “alt-metrics” for evaluating a microbiome project or prospective developments in the microbiome field, both of which are done in the context of existing microbiome big data.

## Abstract (precis)

We introduce two concepts to quantify the novelty of a microbiome. The first, the microbiome novelty score (MNS), allows identification of microbiomes that are especially different from what is already sequenced. The second, the microbiome attention score (MAS), allows identification of microbiomes that have many close neighbors, implying that considerable scientific attention is devoted to their study. By computing a microbiome focus index based on the MNS and MAS, we objectively track and compare the novelty and attention scores of individual microbiome samples and projects over time and predict future trends in the field; i.e., we work toward yielding fundamentally new microbiomes rather than filling in the details. Therefore, MNS, MAS, and MFI can serve as “alt-metrics” for evaluating a microbiome project or prospective developments in the microbiome field, both of which are done in the context of existing microbiome big data.

## INTRODUCTION

With the rapid expansion of microbiome sequencing projects around the globe, relating new data to existing data has become one of the most critical bottlenecks for new studies. High-speed comparison and searching for sample similarities in microbiome data sets have been hindered by the lack of appropriate methods. Well-known analytic platforms, such as mothur (1) and QIIME (2), are optimized to support individual projects but not comparisons and searches across all known microbiomes.

Here, we introduce the Microbiome Search Engine (MSE), which, based on taxonomic similarities, rapidly and precisely identifies for each new microbiome sample the best matches from the extremely large number of known microbiomes. MSE consists of two core modules: a well-organized and regularly updated reference database of microbiomes (the entire Qiita public database [https://qiita.ucsd.edu/], which includes 101,983 curated microbiome samples produced by 293 studies between 2005 and 2017) (Fig. S1 and S2; see also Materials and Methods) and a kernel search algorithm (3, 4) (Fig. S3 and S4; see also Materials and Methods). By generating a real-time, landscape-like view of global microbiome compositions from 16S rRNA amplicon data, MSE provides a readily expandable, generally applicable, and widely assessable approach for knowledge-based microbiome analysis.

10.1128/mBio.02099-18.2 FIG S1 Yearly accumulative curves of total samples, clean samples, and studies from 2005 to 2017 collected by this study. Download FIG S1, JPG file, 0.1 MB. Copyright © 2018 Su et al. 2018 Su et al. This content is distributed under the terms of the Creative Commons Attribution 4.0 International license.

10.1128/mBio.02099-18.3 FIG S2 Accumulative sample curves of 16S rRNA mapping rates. Samples under the red shadow area were dropped due to their low 16S rRNA mapping rate (<80%). Download FIG S2, JPG file, 0.1 MB. Copyright © 2018 Su et al. 2018 Su et al. This content is distributed under the terms of the Creative Commons Attribution 4.0 International license.

10.1128/mBio.02099-18.4 FIG S3 Indexing-based search algorithm of Microbiome Search Engine. (A) Construction of database index; (B) two-tier indexing-based search process. Download FIG S3, JPG file, 0.2 MB. Copyright © 2018 Su et al. 2018 Su et al. This content is distributed under the terms of the Creative Commons Attribution 4.0 International license.

10.1128/mBio.02099-18.5 FIG S4 Microbiome structure reencoding minimizes the memory usage and optimizes the loading cost. (A) For processing the OTU table that contains all samples, typically the whole file is loaded into RAM and then the microbiome structure information is mapped to the right address by querying the hash table. (B) In contrast, MSE reencodes the microbiome features of each reference sample into relative address offsets and directly loads only those candidate samples into RAM by their addresses, which results in a very low loading cost. Download FIG S4, JPG file, 0.2 MB. Copyright © 2018 Su et al. 2018 Su et al. This content is distributed under the terms of the Creative Commons Attribution 4.0 International license.

Tracking the microbiome novelty score (MNS), a metric defined herein based on searching samples against the entire reference database, we detected weak correlation between novelty and alpha-diversity (Spearman *r *<* *0.4). Using this metric, we showed that the structural novelty of the human microbiome is approaching saturation and likely bounded, whereas novelty in environmental habitats remains substantially higher. The microbiome focus index (MFI), derived from the MNS and a microbiome attention score (MAS), can objectively track and compare the structural novelty and received attention scores of individual microbiomes or projects and predict trends in the field. For example, marine and indoor environments and mother-baby interactions could be considered “sleeping beauties” soon to be awakened.

## RESULTS

### Identifying microbiomes with novelty and attention. (i) MNS.

By placing each microbiome sample generated so far in the context of the known microbiome space, MSE provides a bird’s-eye view of the historical development of global microbiome sequencing efforts. We used all 101,983 curated samples to trace the development of microbiome studies captured in the data set from 2010 to 2017 (because the number of samples began to increase rapidly in 2010). The microbiome novelty score (MNS) was proposed to evaluate the compositional uniqueness of a microbiome sample (at the time of its birth) compared to all microbiomes in the database (see Fig. S5 in the supplemental material). With a given sample, *m*, and its top *n* matches, for its match *i*, whose microbiome similarity is *S_i_*, the MNS(*m*) was calculated as indicated below (via Meta-Storms [4] similarity of the top 10 matches [see Materials and Methods]).

(1) $$MNS=1-\frac{\overset{n}{\underset{i=1}{\sum }}\left[S_{i}\times \left(n-i\right)\right]}{\overset{n}{\underset{i=1}{\sum }}\left(n-i\right)}$$

10.1128/mBio.02099-18.6 FIG S5 The microbiome novelty score of a given query sample is calculated based on the similarity values of its top 10 matches. Download FIG S5, JPG file, 0.1 MB. Copyright © 2018 Su et al. 2018 Su et al. This content is distributed under the terms of the Creative Commons Attribution 4.0 International license.

**FIG 1:**
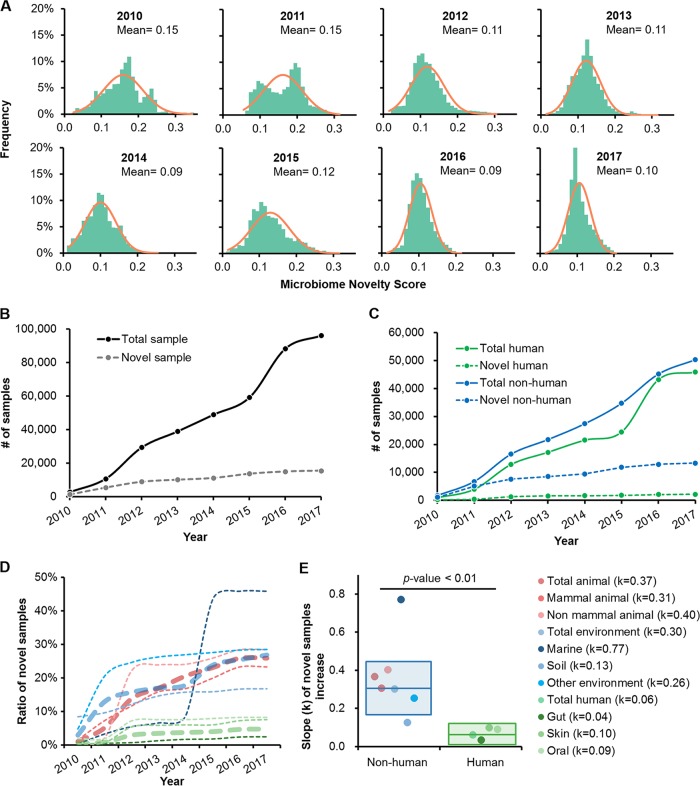
Historical trend of microbiome novelty scores. (A) The MNSs of samples from 2010 to 2017 followed a normal distribution. In each subpanel, the bar chart represents the frequencies of samples and the curve is the simulated standard normal distribution. (B) Yearly accumulative curves of the total numbers of samples and novel samples. From 2010 to 2017, 15,501 samples were identified as novel microbiomes with an MNS of ≥0.15. (C) Yearly accumulative curves of sample numbers for human samples and nonhuman sample. (D) Yearly development of novel sample ratios (defined as the number of novel samples over the number of total samples) in each category. Thick dotted lines represent the ratios of novel samples in high-level categories (human, animal, and natural environments), while thin dotted lines are those in subcategories. (E) Linearly fitting slopes of novel sample ratio increases in each category. The color schemes are the same for panels D and E.

For each microbiome sample, its MNS was derived by searching its sequence against those of all samples produced by past studies (e.g., for a sample published in 2012, its MNS was computed based on its similarity to samples produced prior to 2012). Thus, a higher MNS means lower similarity to those microbiomes that have previously been sampled, suggesting higher novelty. MNS generally followed the normal distribution (Pearson *r *= 0.92 ± 0.07; two-tailed *t* test *P* value = 0.98 ± 0.02, no significant difference [*P* value ≥ 0.01] compared to a simulated normal distribution) (Fig. 1A), suggesting that the number of samples was adequate. The mean of this distribution in the first year of 2010, 0.15, was chosen as the baseline, and samples with an MNS of ≥0.15 were considered novel.

The annual pattern of MNS variation revealed that, although the number of microbiome samples had increased rapidly (there was up to a 36-fold increase from 2010 to 2017), the increase of novelty was much slower (only 10-fold over the same period) (Fig. 1B). In fact, from 2010 to 2017, population-scale studies have continued to resample microbiomes from certain habitats, such as human body sites, causing the unidirectional reduction of mean MNS each year (Fig. 1A). This temporal pattern indicates that current strategies for expanding the boundary of known microbiota is decreasingly efficient, and a new strategy may be required. It is also possible that the natural variation of microbiome compositions is bounded and that the diversity sampled might be approaching saturation.

However, the relationships between sample volume and number of novel samples can vary widely among ecosystems. For example, although the total number of human microbiomes and nonhuman microbiomes were roughly equivalent (*n* = 45,813 versus *n* = 50,268), there were 5-fold-more novel samples from nonhuman habitats than human-derived samples (13,329 versus 2,172) (Fig. 1C). Comparison of the trends of novel samples in each subcategory revealed significantly lower linearly fitting slopes of novel sample ratios (defined as the number of novel samples divided by the number of total samples) (Fig. 1D and Fig. S6) for human samples than for nonhuman samples (two-tailed *t* test *P* value < 0.01) (Fig. 1E). Thus, many more previously unknown microbiome compositions are from environmental habitats than from human-associated ones. Among environmental microbiomes, animal (30.17%, of which 16.75% was mammal contributed), lake (17.97%), marine (15.37%), and soil (10.34%) samples had the most-novel microbiomes; in comparison, among human-associated microbiome compositions, those from the gut, skin, and mouths of humans contributed only 4.07%, 4.71%, and 4.44%, respectively, to the novelty.

10.1128/mBio.02099-18.7 FIG S6 Yearly accumulative curves of total and novel human (top row), environment (middle row), and animal (bottom row) samples. Also shown are those of their subcategories, e.g., gut, oral, and skin within the human category, soil, marine, and other environment within the environment category, and mammal and nonmammal within the animal category. Download FIG S6, JPG file, 0.5 MB. Copyright © 2018 Su et al. 2018 Su et al. This content is distributed under the terms of the Creative Commons Attribution 4.0 International license.

**FIG 2:**
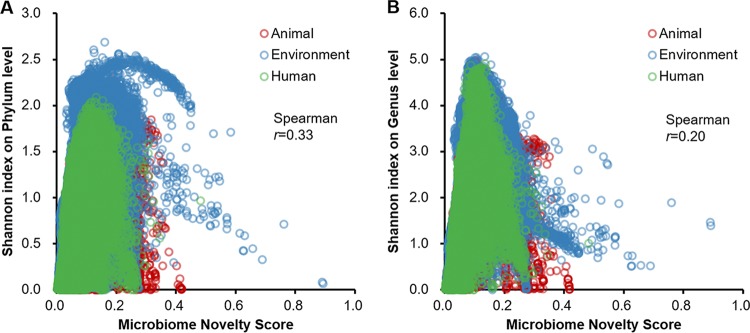
Lack of correlation between the MNSs and Shannon indexes of alpha diversities at both the phylum level (A) and the genus level (B).

In addition, the observed novelty was only weakly associated with the community’s compositional complexity (Fig. 2), as indicated by the low Spearman correlation between the MNS and the Shannon index at the levels of both the phylum (*r *=* *0.33) (Fig. 2A) and the genus (*r *=* *0.20) (Fig. 2B). Furthermore, the MNS was also resistant to variation of amplicon regions of microbiome data, which was verified by the same batch gut samples (*n* = 150) that were amplified from the V1-V3 and V3-V5 regions, respectively (two-tailed *t* test *P* value ≥ 0.01) (Fig. S7 [refer to the supplemental results for details]).

10.1128/mBio.02099-18.8 FIG S7 MNSs of the same gut samples amplified from either the V1-V3 or the V3-V5 region of the 16S rRNA gene. Download FIG S7, JPG file, 0.04 MB. Copyright © 2018 Su et al. 2018 Su et al. This content is distributed under the terms of the Creative Commons Attribution 4.0 International license.

On the other hand, within human habitats, at each of the three major human body sites, the gut, skin, and mouth, the trend in accumulation of novel samples slowed in 2012 and then eventually flattened (slope *k *=* *0.06) (Fig. 1D and Fig. S6). Among the various body sites in humans, despite its highest sample volume, the gut contributes the fewest novel samples compared to the mouth and skin (gut, 631 of 25,936 samples, with a *k *equal to* *0.04; oral, 688 of 8,365 samples, with a *k *equal to* *0.09; skin, 730 of 9,657 samples, with a *k *equal to* *0.10), resulting in the lowest rate of gain in novel samples over the last 5 years (Fig. S6). Notably, for either gut, oral, or skin samples or all human-associated samples, such rates started to enter a more flattened phase in 2012, which was due to the influx of samples from the Human Microbiome Project (5) published in the same year. This underscores the broad and dramatic impact of such systematic studies in expanding the boundary of microbiome novelty. In this way, few novel microbiotas (those with an MNS of ≥0.15) inside or on the human body remain to be discovered, at least in the host populations that are heavily represented at present.

### (ii) MAS.

A parameter based on structural novelty alone is unable to capture the full structural features of a microbiome. A high-MNS sample that spearheads exploration into a new ecosystem can have a high research impact by being subsequently followed by additional sequencing efforts that reveal similar microbiome configurations, or alternatively it can remain “asleep” until such follow-up sequencing ensues. To measure and distinguish such effects, we proposed the microbiome attention score (MAS), which measures the connectivity of a given sample to all subsequent samples in the repository. For a given sample (*m*), its MAS among a total of *n* samples is as follows:

(2) $$\text{MAS}=\overset{n-1}{\underset{i=0,i\neq m}{\sum }}\text{connectivity }\left(m,i\right)$$

where the connectivity to arbitrary sample *i* [connectivity(*m*, *i*)] is defined as the microbiome similarity between samples *m* and *i* (*S_i_*) if *m* is an element of the top *n* matches of *i* and *S_i_* is ≥0.85, or it is 0 if *m* is an element of the top *n* matches of *i*. In other words, MAS is the similarity sum of samples that match sample *m* with a relative high similarity (Meta-Storms similarity ≥ 0.85) (Fig. S8); hence, a higher MAS indicates that more samples with similarity or samples with higher similarity had been sequenced, suggesting higher attention from the scientific community for this input sample. We also set *n* as 10 for consistency in this work.

10.1128/mBio.02099-18.9 FIG S8 The microbiome attention score is calculated based on the number of samples that are related to the given microbiome due to a particular level of similarity. Download FIG S8, JPG file, 0.1 MB. Copyright © 2018 Su et al. 2018 Su et al. This content is distributed under the terms of the Creative Commons Attribution 4.0 International license.

To avoid the possible artificial inflation of MAS (and, thus, MFI), such as that caused by redundant sampling from identical microbiotas, we have implemented the following: (i) all reference samples were collected from Qiita, which contains high-quality microbiome studies with extensive metadata; (ii) duplicate samples with a similarity to the existing reference samples of >99.99% were removed from the reference database; and (iii) when calculating MAS, samples from the same study were excluded.

### (iii) MFI.

**FIG 3:**
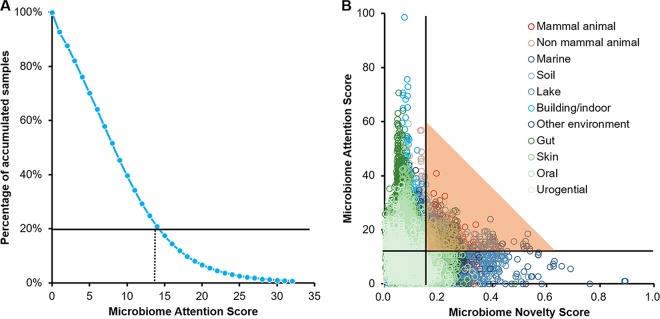
Microbiome attention scores of known microbiome samples. (A) The MAS threshold of 14 is determined based on the top 20% of MAS samples. (B) Distribution of samples by MNS (*x* axis) and MAS (*y* axis). With the cutoff of MNSs was ≥0.15 (novel samples) and that of MASs was ≥14 (high-attention samples), a total number of 2,238 microbiomes were identified as focus samples (dots under the shadows).

We designated the top 20% of the most frequently matched samples by Meta-Storms similarity (corresponding to the threshold MAS of 14) (Fig. 3A) as having high attention among all samples during 2005 to 2017. Hence, samples that have the two attributes of an MNS of ≥0.15 when first sequenced and an MAS of ≥14 were considered “focus” samples (Fig. 3B). A microbiome focus index (MFI), which quantitatively measures the combined novelty and attention of a focus microbiome, is thus calculated as follows:

(3) MFI=MNS×MAS

During the 8 years from 2010 to 2017, 2,238 microbiome samples were identified as having such a focus. The lake (22.29%), animal (22.25%, monkey gut, mouse gut, wild deer gut, dog flea, etc.), marine (15.32%, saline seawater, sponge), soil (14.52%), and human (9.47%, skin, oral, gut, etc.) environments were the environmental types that contributed the most to the highly focused samples. Thus, the MFI derived from the MNS and MAS can serve as a new venue to quantitatively, objectively, and comprehensively evaluate the structural or compositional uniqueness and connectivity of a microbiome among a huge number of samples and studies, which potentially offers advantages to the conventional bibliometric approaches, such as the journal impact factor based on the citation number of the publication, and can be considered an alternative metric (alt-metric) of contribution in the exploration into microbiome space.

### Predicting and tracking the focus microbiome.

From 2010 to 2017, among the total 15,501 novel microbiomes (with an MNS of ≥0.15), only 2,238 were identified as focus samples by equation 3, while others were nonfocus samples, i.e., present in the repository with few connections (with few structurally similarly microbiomes sequenced and with a low MAS). For the focus microbiomes (with both an MNS of ≥0.15 and an MAS of ≥14), from entry into the database, each of them spends a period awaiting discovery by other researchers (during which they have a low MAS), followed by a process of receiving increasing attention (which increases the MAS). Of particular interest are novel samples (with an MNS of ≥0.15) that still have a low MAS (when there are few structurally similar microbiomes) yet have high potential that will be realized only after a certain amount of time has elapsed, i.e., when a large number of structurally similar microbiomes are deposited and their MASs are therefore increased. On the other hand, most microbiomes may never get high attention. Can we predict these “sleeping beauties” that are currently neglected but will later receive high attention from the ∼15,000 microbiomes?

**FIG 4:**
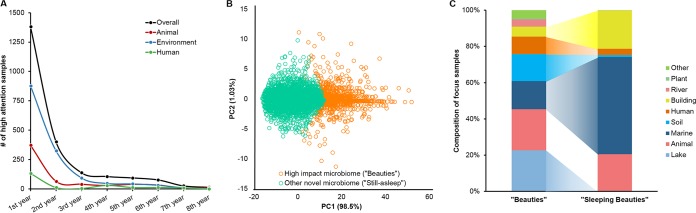
Prediction of sleeping beauty (potential focus) microbiomes. (A) Numbers of focus microbiomes (beauties) that were awaken at the *n*th year after their birth; (B) principal-component analysis of 4-year MASs between beauty samples and still-asleep samples with a random-forest accuracy of 98.78%; (C) habitats of awakened beauties during 2010 to 2017 and of predicted sleeping beauties born since 2015.

Because the MNS is a constant value set when a sample is first published, the key to predicting potential high-focus samples is to identify whether a novel sample would get high attention (i.e., it has an MAS of ≥14) after its birth year. Thus, using historical data, we asked when a novel sample would receive an MAS above threshold. The yearly development curve of focus samples revealed that most known focus samples (90.6%) garnered attention in their first 4 years (Fig. 4A). This result was confirmed by the first 4 years’ MAS pattern of all novel samples produced in 2010 to 2014 (samples after 2015 had only a 3-year MAS); in fact, our random-forest model discriminates focus and nonfocus samples with 98.78% accuracy (Fig. 4B). Based on these results, we built a hybrid model via random-forest regression (see Material and Methods) using the 4-year MASs of novel samples in 2010 to 2014 to predict the sleeping beauties in 2015 to 2017. This model took the <3-year MASs of novel samples as input and estimated their maximum MASs in the future, with a threshold of expected maximum MASs of ≥14 for these potential focus samples.

In contrast to the 2,238 focus samples, the 702 potential focus samples predicted to be activated in the next 4 years were found primarily among marine samples (51.00%, saline seawater, sponges, etc.), building and indoor-environment samples (20.09%, glove surface, water heater, etc.), and animal samples (19.52%, monkey gut, horse gut, mouse gut, etc.), while the proportion of focus samples from lakes and soil were significantly reduced (Fig. 4C; Table 1). Thus, based on recent historical trends, we predict that microbiomes with novel community structures from marine and indoor environments are much more likely to be followed up with additional sampling studies and will become hot spots for microbiome research in the near future until they are sampled as completely as human microbiomes.

**TABLE 1:** Habitats of focus samples, or beauties, during 2010 to 2017 and of predicted potential focus samples, or sleeping beauties, that were born since 2015

Environment	No. of focus samples	No. of predicted focus samples
Lake	499	0
Animal	498	137
Marine	343	358
Soil	325	8
Human	212	23
Building	122	141
River	88	0
Freshwater	39	34
Plant	13	1
Other	99	0

For human microbiomes, the proportion of focus samples decreased from 9.47% to 3.28%, and only 23 samples were considered sleeping beauties (Fig. 4C), mainly due to the now much rarer high-MNS samples from human microbiomes (as shown in Fig. 1C and D). Sixteen of the 23 potential focus microbiomes were sampled from skin in a mother-baby microbial transfer (6). Thus, on the human front, those focusing on mother-baby interactions are predicted to receive extraordinary attention in the next several years.

### Web portal of MSE for computing MNS, MAS, and MFI in real time.

To support online microbiome analysis via MSE, a Web portal is provided at http://mse.single-cell.cn/ (registration or login is not required; see Materials and Methods). For the microbiomes in our database, both metadata (e.g., study description, habitat, sequence type, sampling location and date, etc.) and taxonomical structure are provided for online browsing. When users upload a query microbiome in the form of an operational taxonomic unit (OTU) table, the website returns in real time the matched samples from the database, supplemented with their degrees of similarity, their taxonomic compositions, their MNSs, MASs, and MFIs. In addition, the MNS of the query microbiome is also provided. Furthermore, microbiomes that are of similar taxonomic composition to the query can be downloaded for further analysis.

While the MSE reference database is updated regularly, the MNS of a given microbiome in the reference database remained unchanged with time. This is because, per definition, MNS evaluates the compositional uniqueness of a microbiome sample, at the time of its birth, compared to all microbiomes in the database. In contrast, the MAS is dynamic, since the more structurally similar samples emerge, the higher MAS of a microbiome will be, despite its unchanged MNS. As the product of the MNS and MAS, the MFI is also dynamic. Both the MAS and MFI are updated when the reference database is updated.

## DISCUSSION

Although an enormous volume of large- and small-scale microbiome data sets from various habitats and produced by different studies have been deposited into public data repositories (e.g., HMP [5], EMP [7], and AGP [8]), there are currently few approaches that scale to process and integrate all the microbiome data so that a global view of microbiomes can be generated in real time (9). This has resulted in the majority of microbiome samples being of single use, that is, that suffer from limited data reuse or citations beyond the original scope of the study. Thus, their value depreciated abruptly.

A search-based strategy, such as MSE, which features a microbiome-composition search accelerated up to 3 orders of magnitude relative to the search capabilities of existing strategies (i.e., pairwise comparisons) in databases of 100,000 to 1,000,000 samples, enables such s bird’s-eye view of how each microbiome relates to global microbiomes. For example, by quantitatively defining the novelty of a microbiome, MSE revealed that the novelty of human microbiomes is bounded by their taxonomic dimensions. In fact, efforts expanding the boundaries of the known microbiotas in human (but not nonhuman) habitats has almost reached a plateau, suggesting that a new strategy, such as one focusing on strain- or isolate-level structure or functional variation, might be required.

On the other hand, new metrics, such as the MNS, MAS, and MFI, provide a new way of assessing structural novelty and attention attracted by samples, studies, and areas at a single-microbiome resolution. This quantitative metric, which depends only on the data themselves, may be inherently more accurate and less prone to the influence of unrelated factors than journal or paper citations. Notably, as the definition of the MFI suggests, for focus samples, both the MNS and MAS are important to the MFI. Although no single metric can accurately or thoroughly assess the scientific impact of a microbiome sample, we can argue that focus samples, i.e., those with both high MNSs and high MASs, are likely particularly valuable contributions to our exploration of the microbiome space. For example, by predicting potential focus samples based on the historical evolution of novelty and attention, MSE might potentially help advise policy makers and the scientific community on strategies that efficiently explore the unknown space of microbiome structures. However, appropriate caution should of course be taken against overreliance on any single metric or data source when developing policy.

Finally, MSE is readily expandable, generally applicable and widely assessable. Moreover, because MSE accepts a compositional profile (e.g., OTU, KEGG Orthology, etc.) of a microbiome as search input, the analyses can accommodate both amplicon data sets and metagenomic data sets. We envision that such search against the microbiome database will be an important first step for data analysis at various scales in microbiome studies, just as a BLAST search is essential and universal in sequence analysis studies today.

## MATERIALS AND METHODS

### MSE reference database.

MSE aims to rapidly identify the similar whole-microbiome-level samples of a given query microbiome from a large-scale depository of known microbiomes. The database module, in its present form, consists of the entire Qiita public database (https://qiita.ucsd.edu/), which includes 124,025 public microbiome samples produced by 293 studies (in total) between 2005 to 2017 (see Fig. S1 in the supplemental material), which is regularly updated by adding newly released or published data sets. These studies included the Human Microbiome Project (5), Earth Microbiome Project (7), American Gut Project (8), and other high-impact microbiome studies that cover 18 sampling sources of human-associated habitats, indoor buildings, animal-associated habitats, and various types of natural environments (10–12). Sequences of the 16S rRNA amplicons from the V1-V2, V1-V3, V3-V5, V4, and V6-V9 regions were produced by Illumina HiSeq, MiSeq, or Roche 454 sequencing. After quality control and duplication removal (refer to see “Profiling and normalization” below) (Fig. S2), 101,983 curated microbiome samples were retained for further analysis and interpretation.

### Indexing-based search algorithm of MSE.

The search module of MSE performs a two-tier indexing process (3, 4), as follows. First is the microbiome feature-based dynamic indexing for fast fetch. The dynamic indexing partitions the OTUs on a specified taxonomy level (also referred to as index keys). Therefore, for each sample, the weight of an index key is the sum of relative abundance values that belong to this index key. When constructing the database, MSE precomputes the index keys and their weights for all database samples (Fig. S3A). Then, for a given query microbiome, MSE calculates its index keys in the same way and dynamically selects candidate matches that have the shortest distances to the query on index keys (Fig. S3B). This reduces the time complexity of searching without the loss of match precision.

The second indexing process is whole-microbiome-level similarity computation with structure reencoding-based optimizations. After indexing, MSE identifies the top matches by a pairwise comparison between the query and each of the candidate matches using the Meta-Storms similarity scoring function (4). This algorithm employs a phylogeny-based metric based on the OTUs’ relative abundances to quantitatively assess the similarity between two microbiomes. Typically, the microbiome structures from multiple samples are kept as one centralized file (in BIOM [13], CSV, plaintext, or other equivalent formats) which needs to be entirely loaded into RAM to avoid extra HDD I/O (Hard Disk Drive Input and Output) operations during sample comparison; however, this causes unacceptable memory consumption when 100,000 or more samples are processed (Fig. S4A). To tackle this problem, MSE reencodes the database microbiome structures by sorting the OTUs by relative memory address offsets in the phylogeny tree, so that the community information is directly placed to the right address. Thus, this MSE approach minimizes the loading cost from HDD (Fig. S4B). Furthermore, MSE separates each reference sample’s structure into one individual file that is dispersedly stored in the file system (Fig. S4B). When searching against the whole database, MSE loads only those candidate samples from their particular reencoded files, which maximizes the efficiency of memory usage. Therefore, advanced computing optimization in the two-tier search procedure of MSE greatly and efficiently reduces both the time complexity and the space complexity for large-scale (e.g., over 100,000 samples) microbiome search.

### Profiling and normalization.

All collected samples from Qiita are profiled and annotated by Parallel-META 3 (version 3.4.2) (14) with Greengenes 13-8 (15) on the OTU similarity level of 97%. Variation in 16S rRNA copy number was normalized based on the IMG/M database (16) to maximally reduce the bias of comparison with samples from different platforms and studies. We set the minimum sequence number to 500 and a minimum 16S rRNA mapping rate of 80% for each sample to ensure high quality of the reference data sets (Fig. S2). We also set a threshold of the Meta-Storms similarity of 99.99% to remove duplicated samples. If the similarity between two samples is equal to or higher than the threshold, the one that has a later production/sampling date in the meta-data is dropped. Finally, 101,983 samples passed the quality control and curation (refer to Data Set S1 for study and sample identification numbers).

10.1128/mBio.02099-18.10 DATA SET S1 List of the 101,983 microbiome samples that are used in this study. Detailed information, including sequence files and metadata of these samples, is provided here and can also be assessed via Qiita (http://qiita.ucsd.edu). Download Data Set S1, XLSX file, 4.9 MB. Copyright © 2018 Su et al. 2018 Su et al. This content is distributed under the terms of the Creative Commons Attribution 4.0 International license.

### Construction of a mixed model for maximum MAS estimation using regression and random-forest modeling.

The 4-year MASs of each samples were first normalized by the maximum MAS (MAS_max_) to compute the maximum MAS ratios (MAS/MAS_max_ is always between 0 and 1), and then a regression model was constructed using the maximum MAS ratios of all novel samples between 2010 and 2014 to describe the 4-year development of attention. In this model, the *x* axis represents the year, while the *y* axis represents the expected maximum MAS ratio calculated by the regression. We also computed the random-forest importance of each year’s maximum MAS ratios, and then the maximum MAS of each sample produced after 2015 can be estimated by the following equation:

(4) $${\text{MAS}}_{\text{max}}=\frac{\overset{Y-2014}{\underset{i=1}{\sum }}\left({MAS}_{i}\times \frac{{\text{RF}}_{i}}{{\text{Reg}}_{i}}\right)}{\overset{Y-2014}{\underset{i=1}{\sum }}{\text{RF}}_{i}}$$

Here, *Y* is the samples’ birth year (between 2015 and 2017), MAS*_i_* is the *i*th year’s MAS value, Reg*_i_* is the *i*th year’s maximum MAS ratio calculated by the regression, and RF*i* is the random-forest importance of the *i*th year’s maximum MAS ratio.

### Data availability.

All samples (including the sequence files and metadata) used in this study are available from Qiita (http://qiita.ucsd.edu). Detailed information about samples that passed the quality control check is provided in Data Set S1 in the supplemental material.

### Code availability.

MSE is developed and implemented in C/C++. The indexing and searching algorithm is optimized for parallel computing based on multiple CPUs using the OpenMP library. Both source code and executive binary application packages are available at http://mse.single-cell.cn. With this package, users can build their own reference microbiome databases and perform database searches by any given sample. The search results are compatible with Parallel-META 3 software, so the link between the query sample(s) and searching result(s) can be further mined readily (e.g., analyses of taxonomical diversity, the cooccurrence network, and biomarkers). A means to calculate the microbiome novelty score and microbiome attention score is also included in this package.

### Website portal and online system.

We also provide an online searching engine via a website portal for public use of MSE (http://mse.single-cell.cn). This system accepts input query samples in Parallel-META 3 format and returns search results for both the query sample and matched sample(s) in visualized graphics from multiple perspectives, including a bar chart at the phylum level that shows the comparison of microbiota compositions, the MNS of the query sample derived from all existing database samples, and a result table with similarity values for a matched sample(s) and detailed metadata for in-depth interpretation of the query. A dynamic scheduling strategy is developed and utilized to avoid the task jam for multiple users. In addition, all reference studies and data sets are available for online browsing, and the original sequences and metadata files are also open for download so that user can build their standalone searching environment.[Supplementary-material textS1]

10.1128/mBio.02099-18.1 TEXT S1 Supplemental results. Download Text S1, DOCX file, 0.01 MB. Copyright © 2018 Su et al. 2018 Su et al. This content is distributed under the terms of the Creative Commons Attribution 4.0 International license.
