# Supplementary figures and images for: Identifying and Predicting Novelty in Microbiome Studies
Source: mBio. 2018 Nov 13;9(6):e02099-18. doi: 10.1128/mBio.02099-18 (PMC6234870; doi:10.1128/mBio.02099-18)

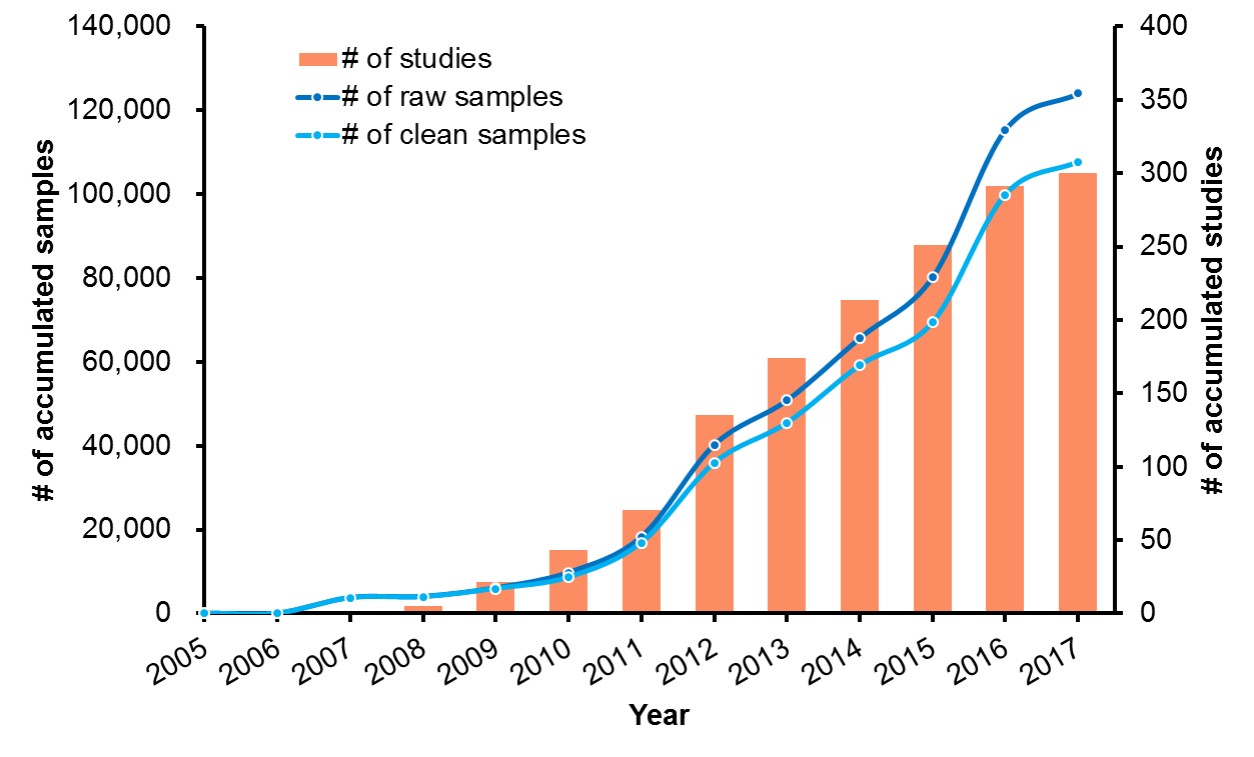

Supplement: FIG S1 [file mbo005184166sf1.jpg]

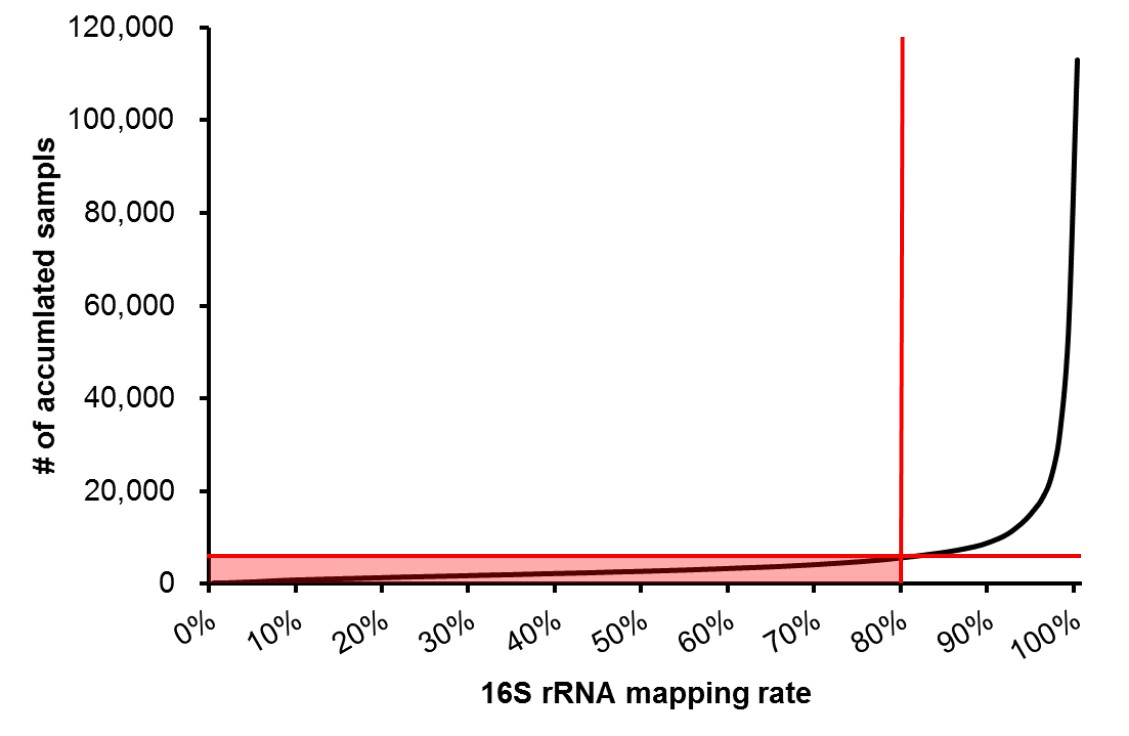

Supplement: FIG S2 [file mbo005184166sf2.jpg]

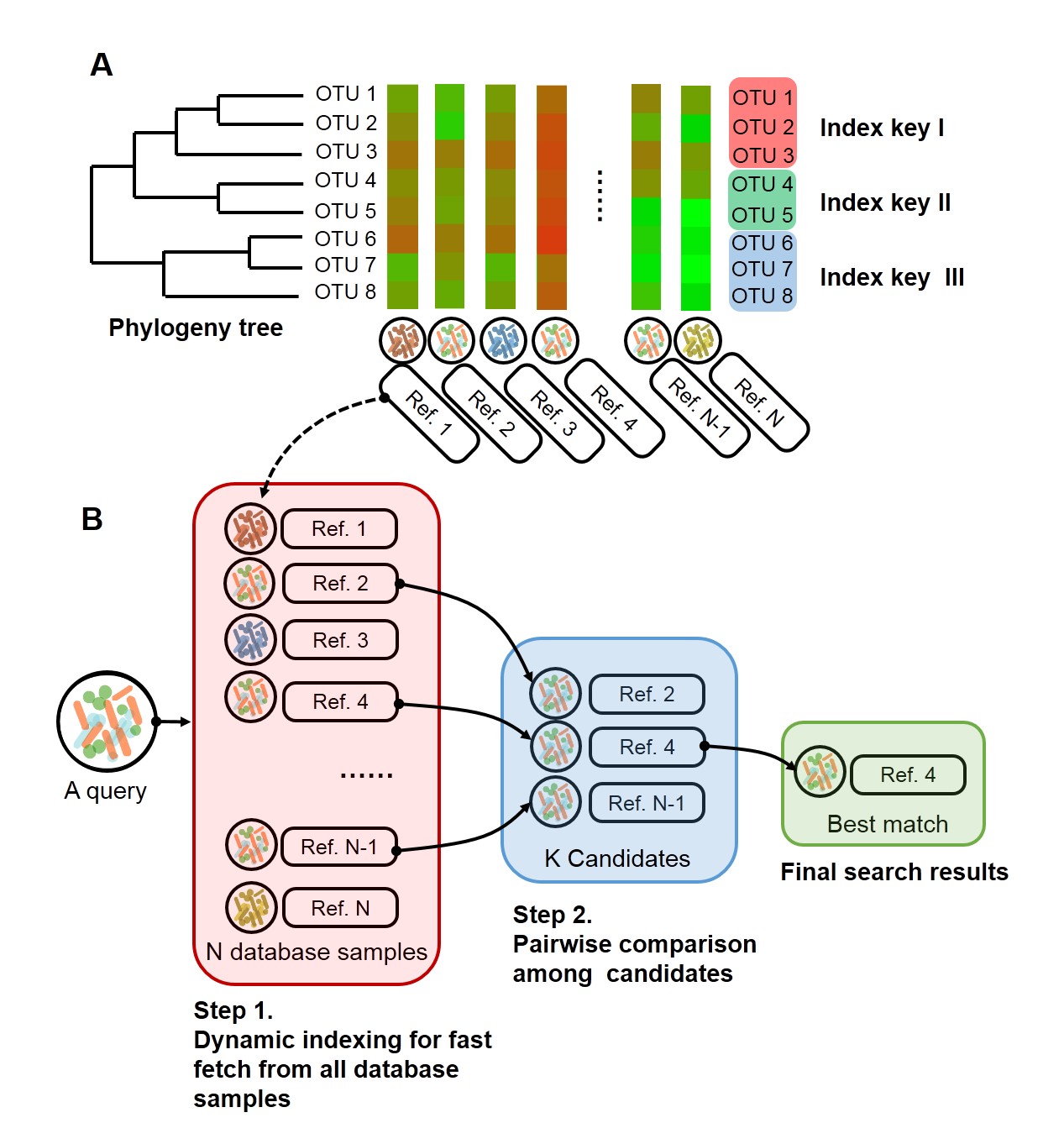

Supplement: FIG S3 [file mbo005184166sf3.jpg]

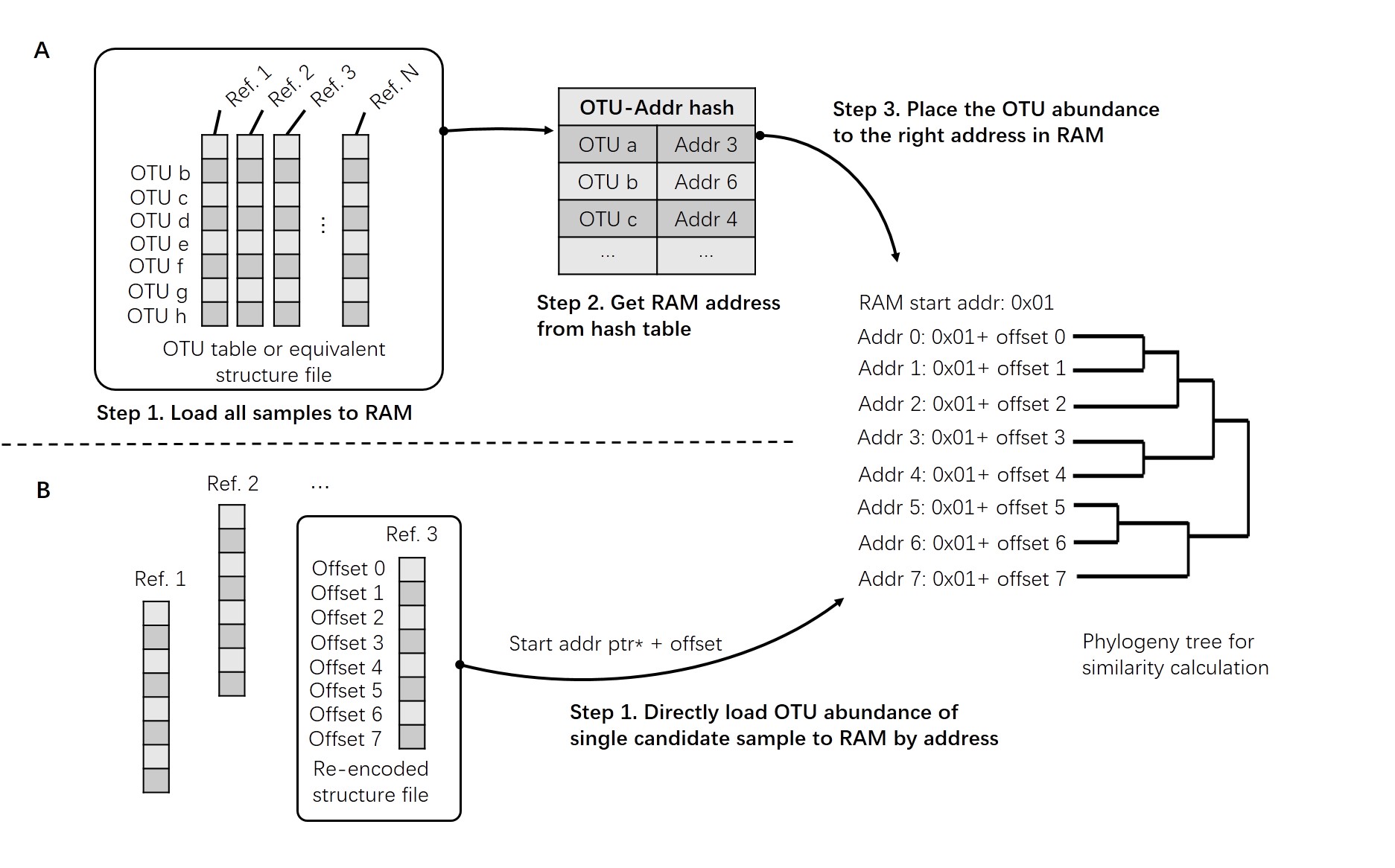

Supplement: FIG S4 [file mbo005184166sf4.jpg]

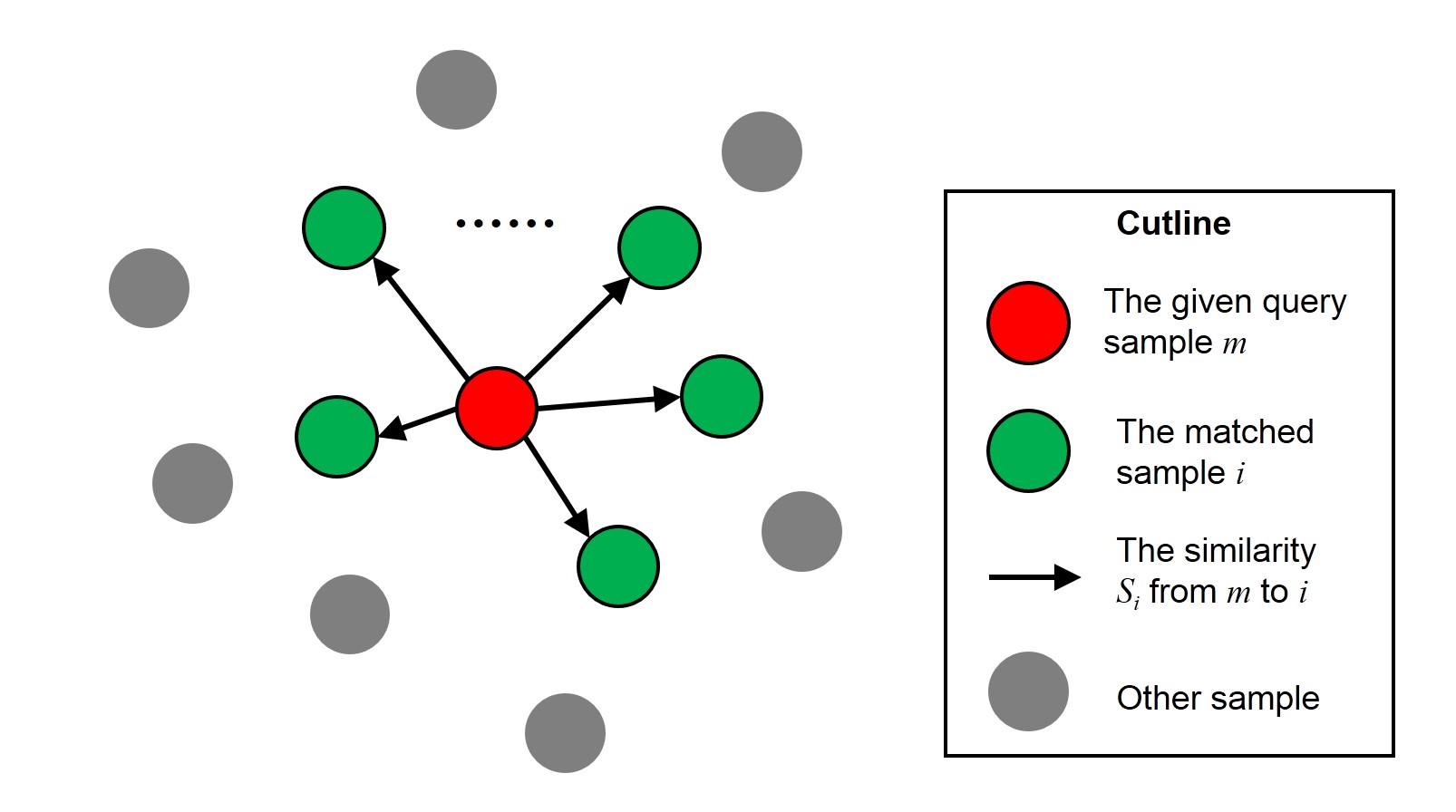

Supplement: FIG S5 [file mbo005184166sf5.jpg]

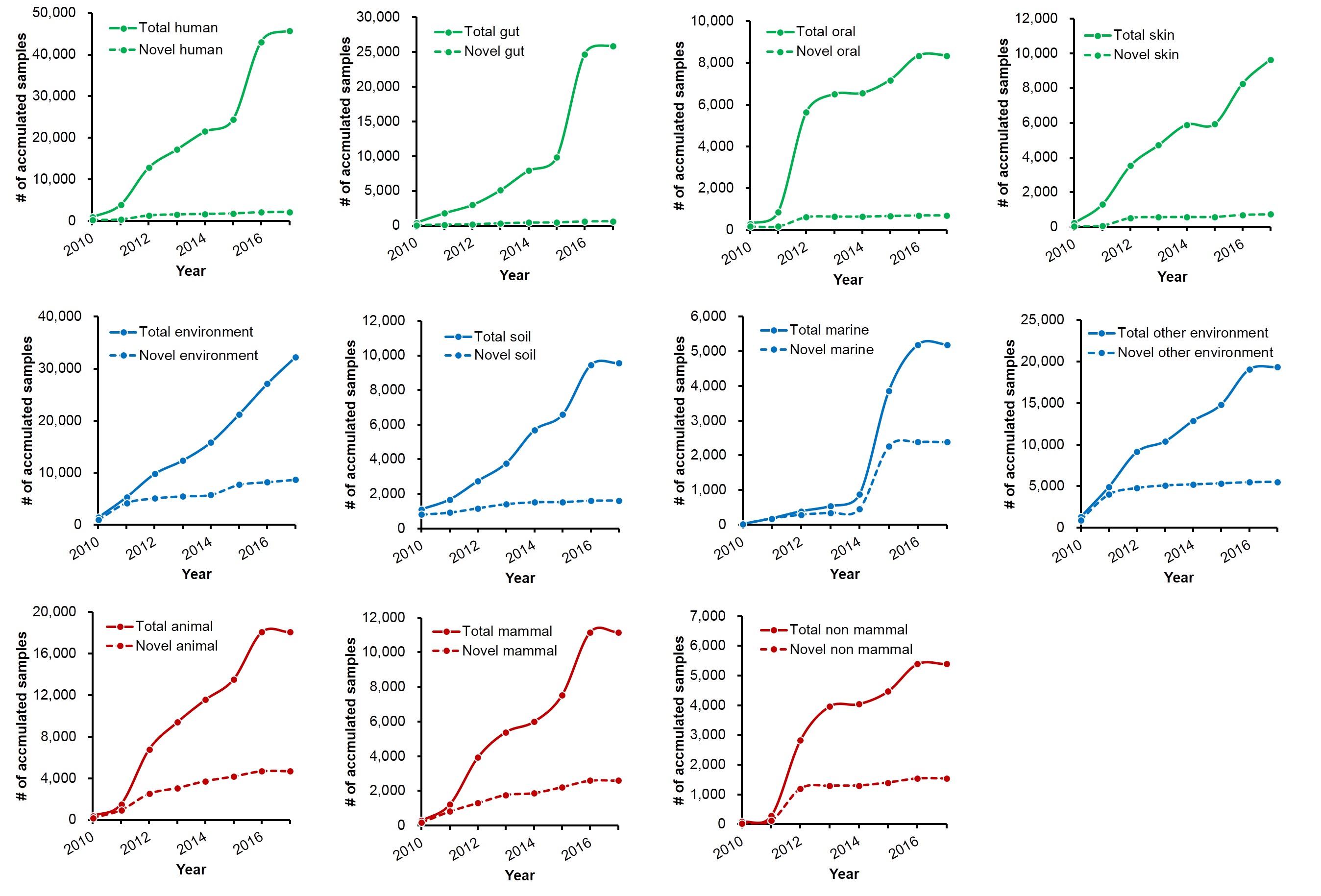

Supplement: FIG S6 [file mbo005184166sf6.jpg]

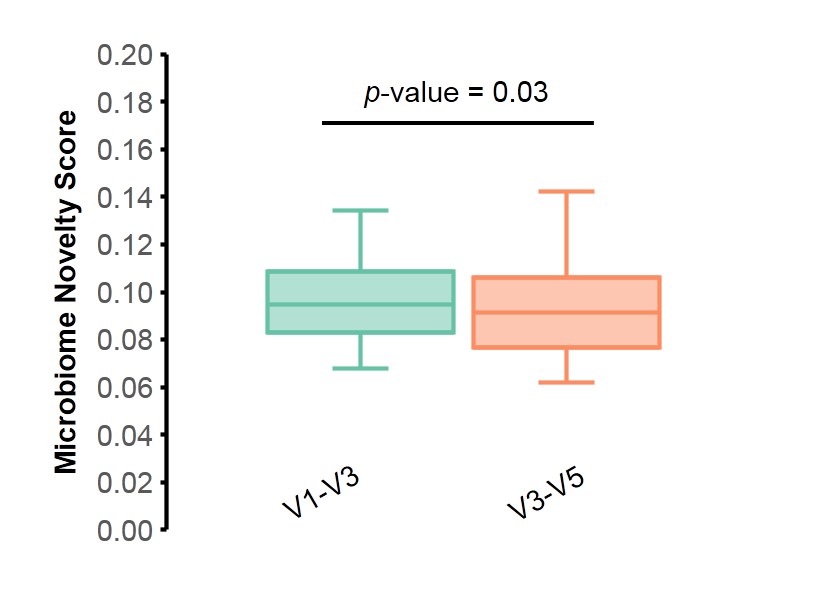

Supplement: FIG S7 [file mbo005184166sf7.jpg]

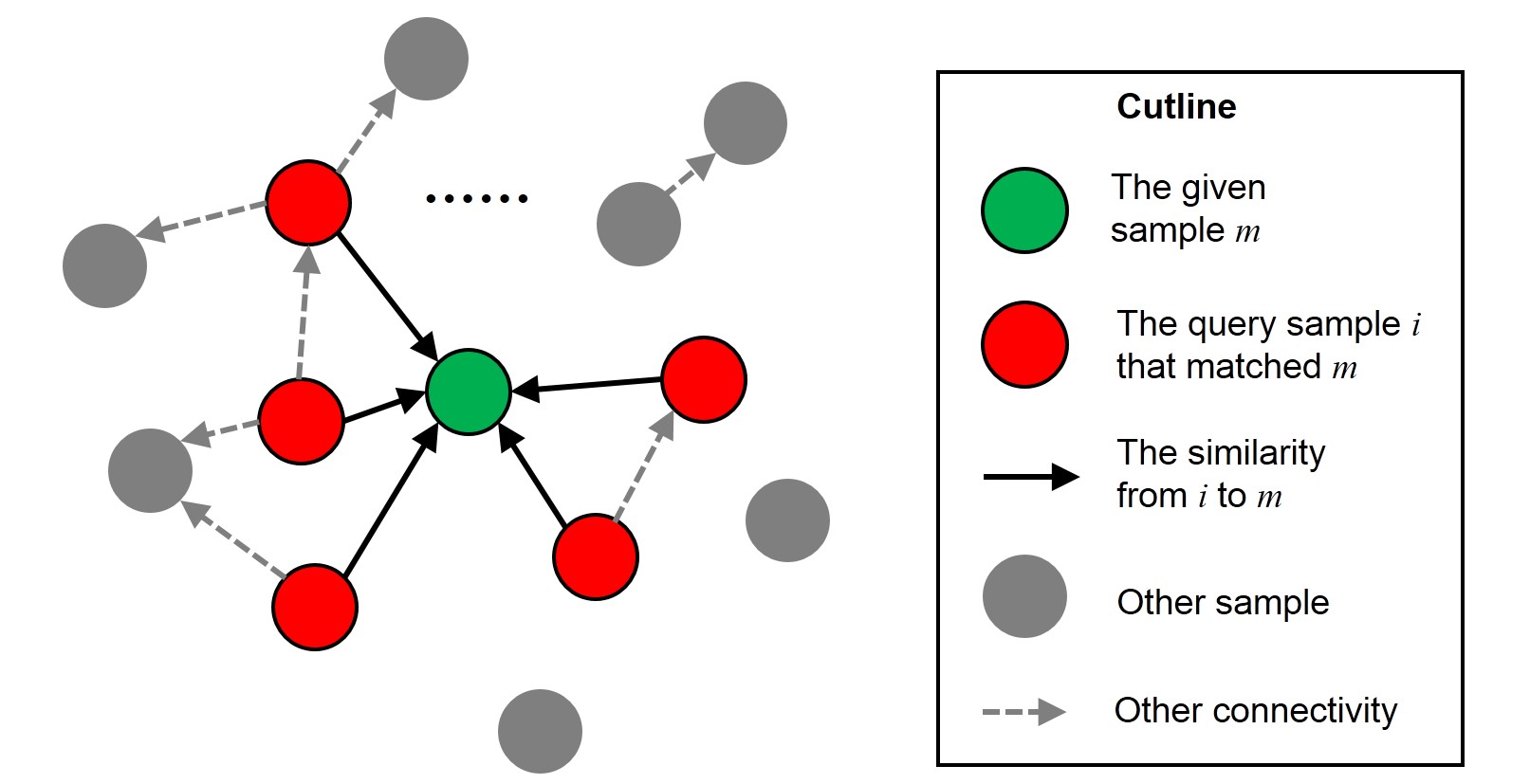

Supplement: FIG S8 [file mbo005184166sf8.jpg]
