# Supplementary material for: Identifying and Predicting Novelty in Microbiome Studies
Source: mBio. 2018 Nov 13;9(6):e02099-18. doi: 10.1128/mBio.02099-18 (PMC6234870; doi:10.1128/mBio.02099-18)
Supplement: TEXT S1 [file mbo005184166s1.docx]

**Identifying and predicting novelty in microbiome studies**

Xiaoquan Su^1,3,4,#^, Gongchao Jing^1,3,4^, Daniel McDonald^2^, Honglei Wang^1,3,4^, Zengbin Wang^1,3,4^, Antonio Gonzalez^2^, Zheng Sun^1,3,4^, Shi Huang^1,3,4^, Jose Navas^2^, Rob Knight^2,#^, Jian Xu^1,2,4,#^

^1^Single-Cell Center, CAS Key Laboratory of Biofuels and Shandong Key Laboratory of Energy Genetics, Qingdao Institute of BioEnergy and Bioprocess Technology, Chinese Academy of Sciences, Qingdao, Shandong, China

^2^Center for Microbiome Innovation, Department of Pediatrics, University of California at San Diego, San Diego, USA

^3^University of Chinese Academy of Sciences, Beijing, China

^4^Laboratory for Marine Biology and Biotechnology, Qingdao National Laboratory for Marine Science and Technology, Qingdao, Shandong, China.

**Supplemental Results**

**Effect of amplicon variation on the calculation of Microbiome Novelty Score.**

Since the MNS is heavily affected by the sampling environment (e.g. natural environment samples have higher MNS than human microbiome samples, **Fig. 1C**, **D** and **E**), to minimize number of variables, we built a benchmark to evaluate the impact of amplicon region on MNS using the same batch of gut samples (*n*=150; produced by Human Microbiome Project Phase I) amplified from either V1-V3 or V3-V5 to evaluate the effect of amplicon region on MNS. No significant difference is observed in the distribution (two-tailed *t*-test *p*-value > 0.01; **Fig. S7**), suggesting MNS values are not significantly affected by the choice of amplified region. Notably, a variable region usually is associated with a preferred sequencing platform. For example, V4-targeted samples are typically sequenced by Illumina, while most V1-V3 and V3-V5 targeted samples are sequenced by 454. Therefore, currently there are few suitable benchmark datasets that would allow quantitative evaluation of the impact of sequencing platform on MNS on a large scale.
